# Formation of a nona­nuclear copper(II) cluster with 3,5-di­methyl­pyrazolate starting from an NHC complex of copper(I) chloride

**DOI:** 10.1107/S2056989020011275

**Published:** 2020-08-21

**Authors:** Christopher A. Dodds, Alan R. Kennedy

**Affiliations:** a Westchem, Department of Pure & Applied Chemistry, University of Strathclyde, 295 Cathedral Street, Glasgow G1 1XL, Scotland

**Keywords:** crystal structure, copper, N-heterocyclic carbene, cluster

## Abstract

The complete nona­nuclear cluster in bis­[1,3-bis­(2,6-di­methyl­phen­yl)imid­azol­ium] di-μ-chlorido-tetra­chlorido­octa­kis­(μ-3,5-di­methyl­pyrazolato)hexa-μ_3_-hydroxido-nona­copper(II) chloro­form disolvate, [HIXy]_2_[Cu_9_(μ-pz*)_8_(μ_3_-OH)_6_(μ_2_-Cl)_2_Cl_4_]·2CHCl_3_ or (C_19_H_21_N_2_)_2_[Cu_9_(C_5_H_7_N_2_)_8_Cl_6_(OH)_6_]·2CHCl_3_, where pz* is the 3,5-di­methyl­pyrazolyl anion, C_5_H_7_N_2_^−^, and HIXy is the 1,3-bis­(2,6-di­methyl­phen­yl)imidazolium cation, C_19_H_21_N_2_^+^, is generated by a crystallographic centre of symmetry with a square-planar Cu^II^ ion bound to four μ_3_-OH ions lying on the inversion centre. Of the four remaining unique Cu^II^ atoms, three adopt CuN_2_O_2_Cl square-pyramidal coordination geometries with the chloride ion in the apical position and one has a distorted CuN_2_OCl tetra­hedral geometry. The dianionic nona­nuclear core can be described as a 24-membered [CuNN]_8_ ring that contains a Cu_9_O_6_Cl_6_ core. The cluster features three intra­molecular O—H⋯Cl hydrogen bonds. In the crystal, weak C—H⋯N and C—H⋯Cl inter­actions link the components. Polynuclear paramagnetic clusters of this type are of considerable inter­est due to their relevance to both the bioinorganic and single-mol­ecule magnets research fields.

## Abstract 2

The complete nona­nuclear cluster in bis­[1,3-bis­(2,6-di­methyl­phen­yl)imidazolium] di-μ-chlorido-tetra­chlorido-octa­kis­(μ-3,5-di­methyl­pyrazolato)hexa-μ_3_-hydroxido-nona­copper(II) chloro­form disolvate, [HIXy]_2_[Cu_9_(μ-pz*)_8_(μ_3_-OH)_6_(μ_2_-Cl)_2_Cl_4_]·2CHCl_3_ or (C_19_H_21_N_2_)_2_[Cu_9_(C_5_H_7_N_2_)_8_Cl_6_(OH)_6_]·2CHCl_3_, where pz* is the 3,5-di­methyl­pyrazolyl anion, C_5_H_7_N_2_^−^, and HIXy is the 1,3-bis­(2,6-di­methyl­phen­yl)imidazolium cation, C_19_H_21_N_2_^+^, is generated by a crystallographic centre of symmetry with a square-planar Cu^II^ ion bound to four μ_3_-OH ions lying on the inversion centre.

## Chemical context   

The study of N-heterocyclic carbene (NHC) complexes of the group 11 metals has proven fruitful for researchers active in this field. Copper (Egbert *et al.*, 2013 ▸) and gold (Díez-González *et al.*, 2009 ▸) complexes have proven particularly useful in catalysis while silver complexes are routinely used as NHC transfer reagents in addition to finding applications as pharmaceutical species (Garrison & Youngs, 2005 ▸). Our inter­est has been the study of the structural chemistry of copper(I) NHC species and, in particular, the replacement of the chloride ligand in [Cu(NHC)Cl] with a variety of pseudohalides, including thio­cyanate and cyanate (Dodds & Kennedy, 2014 ▸; Dodds *et al.*, 2019 ▸). In addition, we have been keen to highlight novel copper(II) species that can form when exploring copper(I) NHC complexes, such as the curious [(1,3-dimesityl-1*H*-imidazol-3-ium-2-yl)methano­lato]copper(II) chloride dimer that formed when formaldehyde was inserted into a copper–carbene bond (Dodds & Kennedy, 2018 ▸).

We sought to extend our studies through the reaction of [Cu(NHC)Cl] with the scorpionate ligand hydro­tris­(3,5-di­methyl­pyrazol­yl)borate (Tp*), hoping to replace the chloride ligand with Tp*. The reaction of [Cu(IXy)Cl] [IXy = 1,3-bis­(2,6-di­methyl­phen­yl)imidazol-2-yl­idene] with an impure batch of NaTp* (predominant contaminant unreacted 3,5-di­methyl­pyrazole) in chloro­form at room temperature resulted in the isolation of a blue solution, which yielded a pale-red powder. Vapour diffusion of diethyl ether into a chloro­form solution of this powder generated both colourless and green crystals. The colourless crystals were analysed by X-ray diffraction and were identified as unreacted [Cu(IXy)Cl].
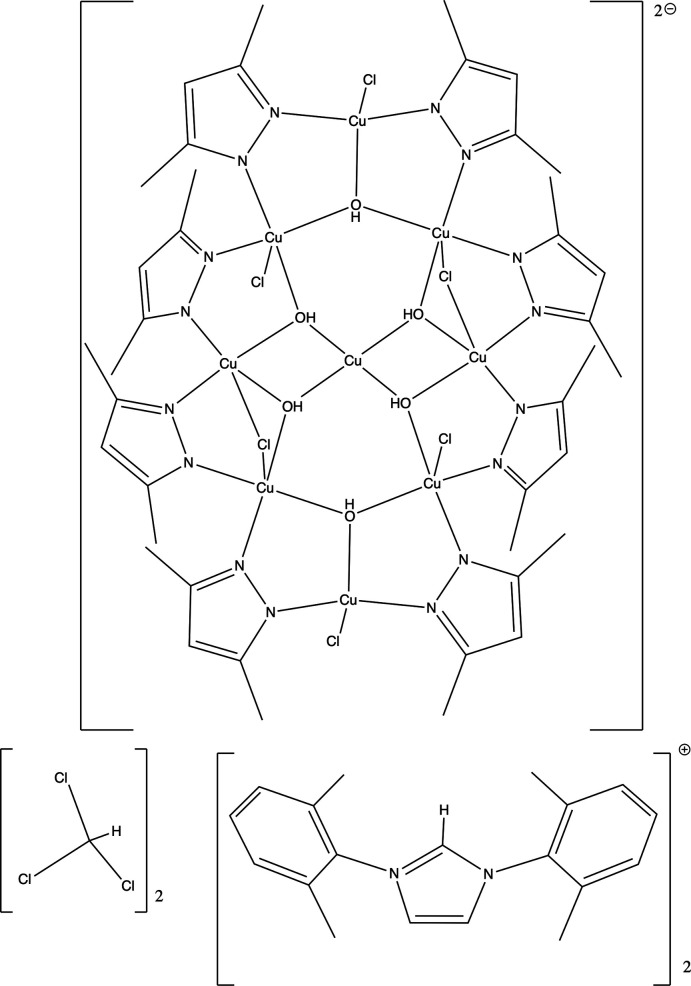


The green crystals were also suitable for X-ray diffraction studies and were identified as the title ionic species [HIXy]_2_ [Cu_9_(μ-pz*)_8_(μ_3_-OH)_6_(μ_2_-Cl)_2_Cl_4_]·2CHCl_3_ (I) (where pz* is 3,5-di­methyl­pyrazolyl, C_5_H_7_N_2_^−^), with the dianion being an unusual nona­nuclear copper(II) cluster. Subsequent attempts to rationally prepare this species have proven unsuccessful to date, and consequently the mechanism for the formation of this species is unknown. There are a large number of examples in the Cambridge Structural Database (CSD) of complexes containing trinuclear triangular μ_3_-OH capped copper(II) clusters (Groom *et al.*, 2016 ▸). On searching the CSD for structures containing a central Cu_9_O_6_ core identical to the structure reported, no exact matches were found. The closest match found was the nona­nuclear Cu^II^ complex [Cu_9_(*L*)_4_(μ_3_-OH)_4_(MeOH)_2_] (*L* = penta­dentate trianionic Schiff-base ligand with N_2_O_3_ donor atoms) (Khanra *et al.*, 2009 ▸). This complex consists of a central copper(II) atom, which resides in a Jahn–Teller-distorted octa­hedral geometry, coordinated by six oxygen atoms. The remaining Cu^II^ atoms are in distorted square-based pyramidal coordination environments, with each Cu^II^ ion coordinated by one nitro­gen atom and four oxygen atoms. The imidazolium cation, [HIXy]^+^, has been structurally characterized previously, with two entries in the CSD (Ilyakina *et al.*, 2012 ▸; Bortoluzzi *et al.*, 2016 ▸).

## Structural commentary   

**Figure 1:**
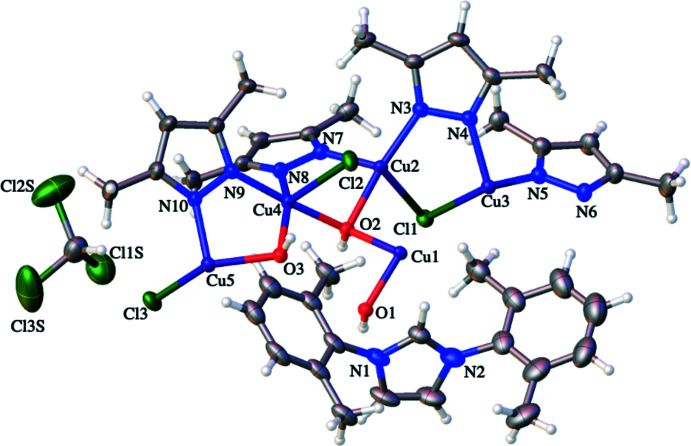
Contents of the asymmetric unit of (I) with non-H atoms shown as 50% probability ellipsoids and H atoms as spheres of arbitrary size.

**Figure 3:**
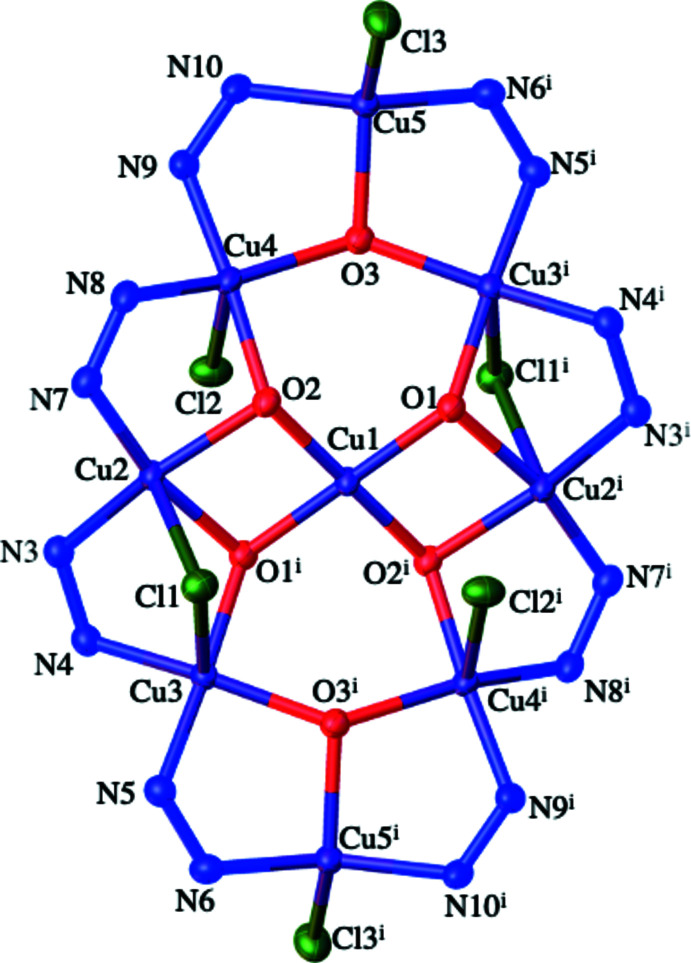
Simplified diagram of the coordination bonds within the anion in (I). The outer ring is a 24-membered [CuN_2_]_8_ unit that contains a Cu- and O-based core.

The mol­ecular structure of (I) consists of a nona­nuclear dianion and two imidazolium cations: two solvent CHCl_3_ mol­ecules complete the structure. The dianion is crystallographically centrosymmetric (*Z*′ = 0.5) with Cu1 occupying the centre of symmetry. The dianion can thus be best thought of as two [Cu_4_(μ-pz*)_4_(μ_3_-OH)_3_(μ_2_-Cl)Cl_2_] moieties with each connected to a Cu^II^ centre *via* two μ_3_-OH groups (Figs. 1 ▸ and 2 ▸). This central Cu^II^ ion resides in a square-planar geometry, as evidenced by the O—Cu1—O bond angles (Table 1 ▸). The eight outer Cu^II^ ions are found in two different coordination environments. Cu5 and Cu5^i^ [symmetry code: (i) –*x*, –*y*, –*z*] can be described as residing in flattened tetra­hedral geometries (sum of bond angles = 666.32°) and each of these Cu centres bonds to a single N atom of each of two pz* ligands, to one μ_3_-OH ligand and to a terminal chloride ligand. The N—Cu—N and O—Cu—Cl bond angles have widened to 150.43 (14) and 133.07 (7)°, respectively, with the remaining angles compressed to between 92.56 (10) and 98.93 (9)°, see Table 1 ▸. The Cu5—O3 bond length is 2.029 (2) Å, which is similar to the values of other reported Cu—O bond lengths between Cu^II^ ions and μ_3_-OH groups (Casarin *et al.*, 2005 ▸; Khanra *et al.*, 2009 ▸). The two Cu5—N bond lengths are statistically identical at 1.924 (3) and 1.927 (3) Å and finally the Cu5—Cl bond length is 2.2466 (19) Å. The remaining six Cu^II^ centres (Cu2, Cu3 and Cu4 and their symmetry clones) reside in distorted square-based pyramidal geometries. Each of these metal ions is coordinated to a single N atom from each of two pz* ligands, to two μ_3_-OH ligands and to a chloride ligand (either terminal or bridging). The *cis*-N_2_O_2_ basal planes are comprised of the μ_3_-hydroxo oxygen atoms and pz* nitro­gen atoms with the chloride ligands occupying the apical positions. Details of coordination bond lengths and angles are given in Table 1 ▸, with some pertinent features highlighted below. The Cu—N bond length range is 1.943 (3) to 1.979 (3) Å while the Cu—O bond length range is 1.986 (2) to 2.115 (2) Å with both sets of values comparing well to previously reported examples of multinuclear copper(II) complexes containing both μ_3_-OH groups and pyrazolate ligands (Casarin *et al.*, 2005 ▸). The Cu—Cl bond lengths vary as expected, depending on whether the chloride is bonding *via* bridging or terminal modes. The Cu4—Cl2 bond length for the terminal chloride anion is 2.5191 (9) Å while the bridging chloride ions have longer Cu—Cl bond lengths of 2.5755 (8) and 2.6282 (9) Å. Note that all these Cu—Cl and Cu—N distances are longer than those found for four-coordinate Cu5, but that the Cu5—O3 distance fits within the range given above. These inter­actions combine to give a nona­nuclear dianion whose core can be envisioned as a linear Cu(O)_2_Cu(O)_2_Cu unit subtended by two Cu_3_O units (Figs. 3 ▸, 4 ▸ and 5).

Of the μ_3_-OH groups, atom O3 is situated 0.364 (2) Å out of the plane defined by the three copper atoms (Cu3^i^/Cu4/Cu5) whilst O1 and O2 adopt more pyramidal geometries and are situated out of the planes defined by the copper atoms (Cu1^i^/Cu2^i^/Cu3^i^ and Cu1/Cu2/Cu4) by 0.651 (2) and 0.758 (2) Å, respectively.

The structural parameters of the imidazolium cation, [HIXy]^+^, in (I) compare well to the previously reported structures (Ilyakina *et al.*, 2012 ▸; Bortoluzzi *et al.*, 2016 ▸). The C1—N bond lengths of the heterocycle are slightly shorter at 1.322 (5) and 1.334 (5) Å compared to the 1.333–1.357 Å range in the previously reported structures. The N1—C1—N2 bond angle of the heterocycle is 109.5 (3)° compared to 108.6° for both of the previously reported structures.

## Supra­molecular features   

Table 2 ▸ shows the short hydrogen-bonding contacts of the structure. All three classical hydrogen bonds are intra­molecular O—H⋯Cl contacts and the inter­molecular contacts are thus non-classical inter­actions involving C atoms. The main inter­actions observed between the anion and the cation involve the labile C1—H1 group of the imidazolium cation. This inter­acts with two Cl ligands of the anion through the C1—H1⋯Cl1 hydrogen bond and through a π geometry C—H to Cl3^i^ inter­action [C⋯Cl = 3.093 (2) Å]. The other inter­actions of Table 2 ▸ are all inter­nal to the [HIXy]_2_ [Cu_9_(μ-pz*)_8_(μ_3_-OH)_6_(μ_2_-Cl)_2_Cl_4_]·2CHCl_3_ unit, except for the C2—H2⋯Cl3^ii^ contact [symmetry code: (ii) –*x* + 1, –*y*, –*z*]. This short contact exists between an H atom of the unsaturated backbone of the imidazolium cation and a chloride ligand of a neighbouring anion and connects anions and cations by translation along the *a*-axis direction.

## Database survey   

Outside the complex reported herein there are eleven structures reported in the CSD (Version 5.41, update no. 1, March 2020; Groom *et al.*, 2016 ▸) that contain a Cu_9_O_6_ core as observed in the complex reported. Of these, only one structure is truly a nona­nuclear copper(II) cluster (Khanra *et al.*, 2009 ▸: refcode DUGLOH). There are two reports in the CSD of structures that contain the imidazolium cation [HIXy]^+^ (Ilyakina *et al.*, 2012 ▸: refcode ZEFBAP; Bortoluzzi *et al.*, 2016 ▸: refcode QAJTIH).

## Synthesis and crystallization   

[Cu(IXy)Cl] (234 mg, 0.625 mmol) was dissolved in chloro­form (5 ml) and NaTp* (200 mg, 0.625 mmol) dissolved in chloro­form (5 ml) was added. (Retrospectively it was found that the NaTp* used was not pure, containing significant qu­anti­ties of unreacted 3,5-di­methyl­pyrazole.) The initially pale-yellow solution turned pale green and the solution was left stirring for 24 h. After this time, the solution had turned blue and it appeared as though a small amount of white precipitate had formed. The mixture was filtered through Celite and the solvent was removed *in vacuo*. During the removal of the solvent, the colour changed from blue to deep red–brown, resulting in the isolation of a deep red–brown oil. Diethyl ether was added, which resulted in the precipitation of a pale-red solid, which was isolated by filtration and dried, yielding 180 mg of solid. In an effort to grow crystals suitable for single-crystal X-ray diffraction studies, 19 mg of solid was dissolved in chloro­form (0.5 ml) and vapour diffused with diethyl ether. The majority of the crystals that formed were colourless and analysed as unreacted [Cu(IXy)Cl]. The green crystals that were isolated analysed as the reported [HIXy]_2_[Cu_9_(μ-pz*)_8_(μ_3_-OH)_6_(μ_2_-Cl)_2_Cl_4_]·2CHCl_3_.

## Refinement   

Crystal data, data collection and structure refinement details are summarized in Table 3 ▸. Data were measured by the EPSRC National Crystallography Service (Coles & Gale, 2012 ▸). All H atoms bound to C were geometrically placed and modelled in riding mode with C—H distances of 0.95, 0.98 and 1.00 Å for *sp* ^2^ CH, methyl, and *sp* ^3^ CH groups, respectively. For methyl groups, the constraint *U*_iso_(H) = 1.5*U*_eq_(C) was applied and elsewhere *U*_iso_(H) = 1.2*U*_eq_(C). The H atoms of the OH groups were positioned as found in a difference map and refined isotropically with the O—H distance restrained to 0.88 (1) Å. Displacement ellipsoids show a relatively high amount of motion in the Cl atoms of the solvent CHCl_3_ mol­ecule, and the highest residual electron density lies close to this feature. Disordered models were constructed, but were not as satisfactory as the ordered model presented.

## Supplementary Material

Crystal structure: contains datablock(s) I, global. DOI: 10.1107/S2056989020011275/hb7934sup1.cif

Structure factors: contains datablock(s) I. DOI: 10.1107/S2056989020011275/hb7934Isup2.hkl

CCDC reference: 2023764

Additional supporting information:  crystallographic information; 3D view; checkCIF report

## Figures and Tables

**Figure 2:**
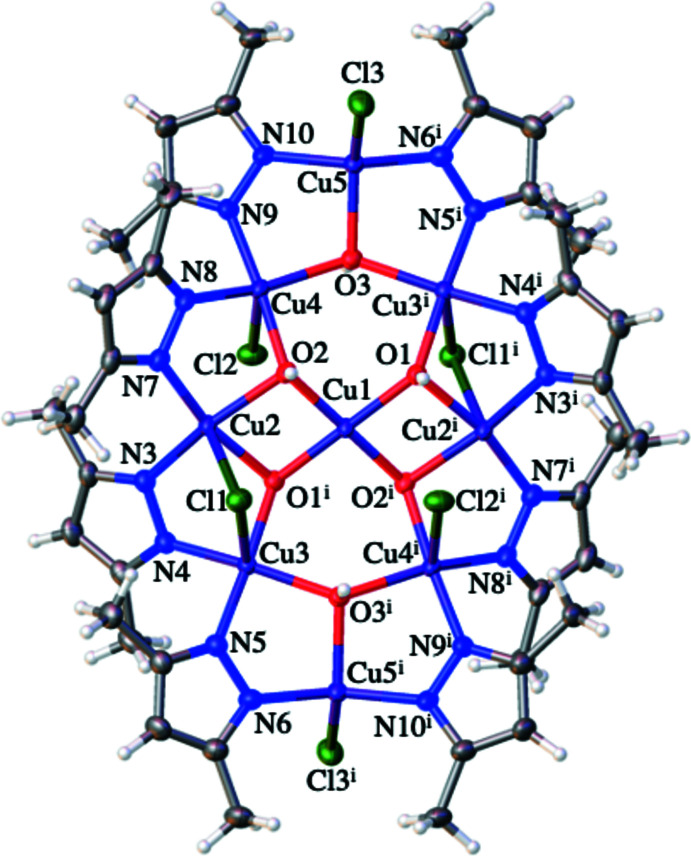
Structure of the centrosymmetric nona­nuclear anion in (I). The symmetry-equivalent atoms are generated by the symmetry operation –*x*, –*y*, –*z*.

**Figure 4:**
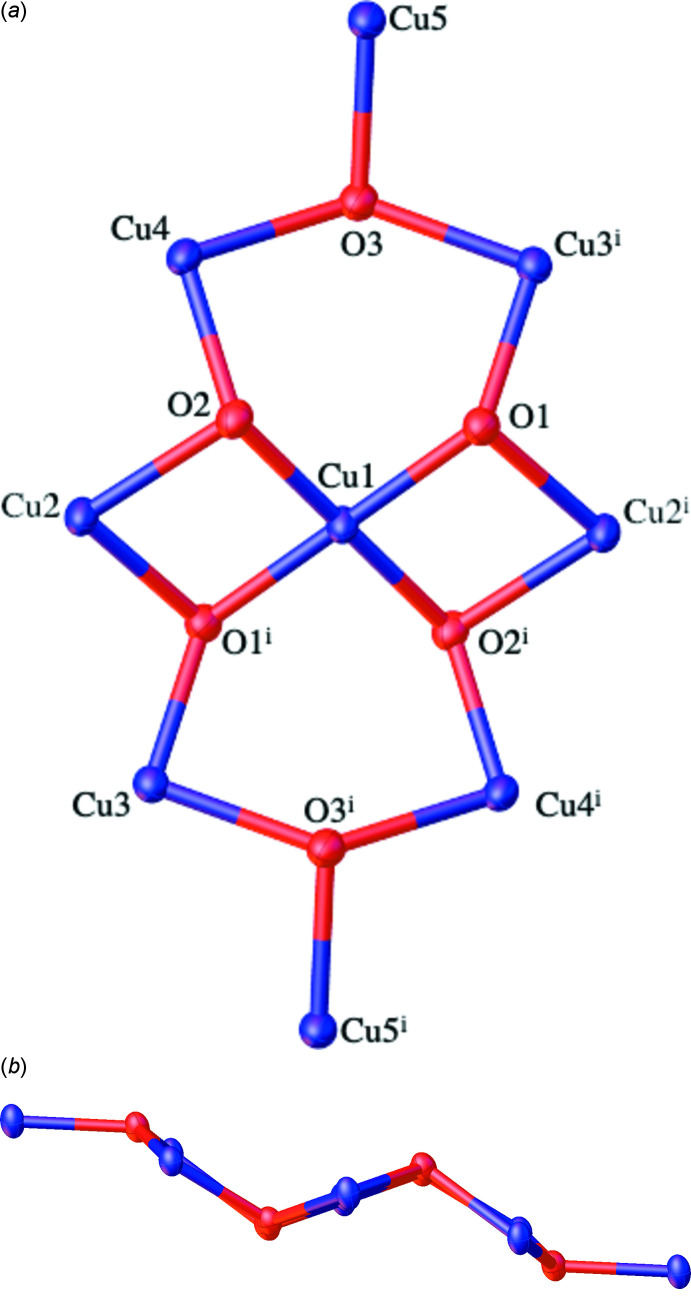
Central Cu_9_O_6_ core in (I) viewed (*a*) from above and (*b*) from the side.

**Table 1:** Selected geometric parameters (Å, °)

Cu1—O1^i^	1.924 (2)	Cu3—O3^i^	2.057 (2)
Cu1—O1	1.924 (2)	Cu3—Cl1	2.5755 (8)
Cu1—O2	1.929 (2)	Cu4—N9	1.943 (3)
Cu1—O2^i^	1.929 (2)	Cu4—N8	1.977 (3)
Cu2—N7	1.947 (3)	Cu4—O2	1.996 (2)
Cu2—N3	1.964 (3)	Cu4—O3	2.115 (2)
Cu2—O1^i^	2.031 (2)	Cu4—Cl2	2.5191 (9)
Cu2—O2	2.044 (2)	Cu5—N6^i^	1.924 (3)
Cu2—Cl1	2.6282 (9)	Cu5—N10	1.927 (3)
Cu3—N5	1.950 (3)	Cu5—O3	2.029 (2)
Cu3—N4	1.979 (3)	Cu5—Cl3	2.2466 (9)
Cu3—O1^i^	1.986 (2)		
			
O1^i^—Cu1—O1	180.0	O1^i^—Cu3—O3^i^	92.81 (9)
O1^i^—Cu1—O2	87.68 (9)	N5—Cu3—Cl1	96.83 (8)
O1—Cu1—O2	92.32 (9)	N4—Cu3—Cl1	101.02 (8)
O1^i^—Cu1—O2^i^	92.32 (9)	O1^i^—Cu3—Cl1	81.45 (6)
O1—Cu1—O2^i^	87.68 (9)	O3^i^—Cu3—Cl1	99.01 (6)
O2—Cu1—O2^i^	180.0	N9—Cu4—N8	93.34 (12)
N7—Cu2—N3	106.25 (11)	N9—Cu4—O2	176.28 (11)
N7—Cu2—O1^i^	167.65 (10)	N8—Cu4—O2	83.51 (10)
N3—Cu2—O1^i^	85.78 (10)	N9—Cu4—O3	87.28 (10)
N7—Cu2—O2	85.87 (10)	N8—Cu4—O3	157.45 (10)
N3—Cu2—O2	165.28 (10)	O2—Cu4—O3	96.39 (9)
O1^i^—Cu2—O2	81.83 (8)	N9—Cu4—Cl2	97.75 (9)
N7—Cu2—Cl1	101.13 (9)	N8—Cu4—Cl2	112.62 (8)
N3—Cu2—Cl1	98.45 (8)	O2—Cu4—Cl2	81.71 (6)
O1^i^—Cu2—Cl1	79.34 (7)	O3—Cu4—Cl2	89.57 (6)
O2—Cu2—Cl1	87.07 (7)	N6^i^—Cu5—N10	150.43 (14)
N5—Cu3—N4	95.03 (11)	N6^i^—Cu5—O3	93.11 (10)
N5—Cu3—O1^i^	177.04 (10)	N10—Cu5—O3	92.56 (10)
N4—Cu3—O1^i^	82.97 (10)	N6^i^—Cu5—Cl3	98.22 (9)
N5—Cu3—O3^i^	89.83 (10)	N10—Cu5—Cl3	98.93 (9)
N4—Cu3—O3^i^	158.66 (10)	O3—Cu5—Cl3	133.07 (7)

Symmetry code: (i) 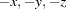.

**Table 2:** Hydrogen-bond geometry (Å, °)

*D*—H⋯*A*	*D*—H	H⋯*A*	*D*⋯*A*	*D*—H⋯*A*
O1—H1*H*⋯Cl2^i^	0.87 (1)	2.68 (4)	3.098 (2)	111 (3)
O2—H2*H*⋯Cl1	0.88 (1)	2.95 (5)	3.246 (2)	102 (3)
O3—H3*H*⋯Cl2	0.88 (1)	2.81 (4)	3.277 (2)	115 (3)
C1—H1⋯Cl1	0.95	2.48	3.336 (4)	151
C2—H2⋯Cl3^ii^	0.95	2.71	3.467 (4)	138
C1*S*—H1*S*⋯Cl3	1.00	2.51	3.395 (5)	147
C20—H20*A*⋯N7	0.98	2.60	3.477 (5)	149
C24—H24*C*⋯N5	0.98	2.55	3.307 (5)	134
C25—H25*A*⋯Cl1	0.98	2.77	3.652 (4)	150
C29—H29*A*⋯Cl3^i^	0.98	2.81	3.572 (5)	135
C35—H35*A*⋯Cl2	0.98	2.90	3.764 (4)	148
C39—H39*A*⋯Cl3	0.98	2.80	3.644 (4)	144

Symmetry codes: (i) 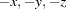; (ii) 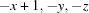.

**Table 3:** Experimental details

Crystal data	
Chemical formula	(C_19_H_21_N_2_)_2_[Cu_9_(C_5_H_7_N_2_)_8_Cl_6_(OH)_6_]·2CHCl_3_
*M*_r_	2441.10
Crystal system, space group	Triclinic, *P* 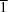
Temperature (K)	100
*a*, *b*, *c* (Å)	12.9974 (9), 13.9305 (10), 14.7162 (10)
α, β, γ (°)	106.143 (3), 93.254 (2), 99.819 (2)
*V* (Å^3^)	2506.6 (3)
*Z*	1
Radiation type	Mo *K*α
μ (mm^−1^)	2.25
Crystal size (mm)	0.19 × 0.08 × 0.05

Data collection	
Diffractometer	Rigaku AFC12 Saturn724+ CCD
Absorption correction	Multi-scan (*CrystalClear*; Rigaku, 2012 ▸)
*T*_min_, *T*_max_	0.593, 1.000
No. of measured, independent and observed [*I* > 2σ(*I*)] reflections	37232, 11446, 10024
*R*_int_	0.055
(sin θ/λ)_max_ (Å^−1^)	0.651

Refinement	
*R*[*F* ^2^ > 2σ(*F* ^2^)], *wR*(*F* ^2^), *S*	0.048, 0.137, 1.04
No. of reflections	11446
No. of parameters	598
No. of restraints	3
H-atom treatment	H atoms treated by a mixture of independent and constrained refinement
Δρ_max_, Δρ_min_ (e Å^−3^)	1.28, −1.39

Computer programs: *CrystalClear-SM Expert* (Rigaku, 2012 ▸), *SHELXS97* (Sheldrick, 2008 ▸), *SHELXL2014* (Sheldrick, 2015 ▸), *Mercury* (Macrae *et al.*, 2020 ▸) and *OLEX2* Dolomanov *et al.*, 2003 ▸).

## supplementary crystallographic information

### Crystal data

**Table:**

(C_19_H_21_N_2_)_2_[Cu_9_(C_5_H_7_N_2_)_8_Cl_6_(OH)_6_]·2CHCl_3_	*Z* = 1
*M**_r_* = 2441.10	*F*(000) = 1239
Triclinic, *P*1	*D*_x_ = 1.617 Mg m^−^^3^
*a* = 12.9974 (9) Å	Mo *K*α radiation, λ = 0.71073 Å
*b* = 13.9305 (10) Å	Cell parameters from 35050 reflections
*c* = 14.7162 (10) Å	θ = 2.3–27.5°
α = 106.143 (3)°	µ = 2.25 mm^−^^1^
β = 93.254 (2)°	*T* = 100 K
γ = 99.819 (2)°	Block, green
*V* = 2506.6 (3) Å^3^	0.19 × 0.08 × 0.05 mm

### Data collection

**Table:**

Rigaku AFC12 Saturn724+ CCD diffractometer	10024 reflections with *I* > 2σ(*I*)
Radiation source: sealed tube	*R*_int_ = 0.055
profile data from ω–scans	θ_max_ = 27.6°, θ_min_ = 3.5°
Absorption correction: multi-scan (CrystalClear; Rigaku, 2012)	*h* = −16→16
*T*_min_ = 0.593, *T*_max_ = 1.000	*k* = −17→18
37232 measured reflections	*l* = −19→19
11446 independent reflections	

### Refinement

**Table:**

Refinement on *F*^2^	Primary atom site location: structure-invariant direct methods
Least-squares matrix: full	Hydrogen site location: mixed
*R*[*F*^2^ > 2σ(*F*^2^)] = 0.048	H atoms treated by a mixture of independent and constrained refinement
*wR*(*F*^2^) = 0.137	*w* = 1/[σ^2^(*F*_o_^2^) + (0.0746*P*)^2^ + 4.3606*P*] where *P* = (*F*_o_^2^ + 2*F*_c_^2^)/3
*S* = 1.04	(Δ/σ)_max_ < 0.001
11446 reflections	Δρ_max_ = 1.28 e Å^−^^3^
598 parameters	Δρ_min_ = −1.39 e Å^−^^3^
3 restraints	

### Special details

**Table:**

Geometry. All esds (except the esd in the dihedral angle between two l.s. planes) are estimated using the full covariance matrix. The cell esds are taken into account individually in the estimation of esds in distances, angles and torsion angles; correlations between esds in cell parameters are only used when they are defined by crystal symmetry. An approximate (isotropic) treatment of cell esds is used for estimating esds involving l.s. planes.

### Fractional atomic coordinates and isotropic or equivalent isotropic displacement parameters (Å^2^)

**Table:**

	*x*	*y*	*z*	*U*_iso_*/*U*_eq_	
Cu1	0.0000	0.0000	0.0000	0.01912 (12)	
Cu2	−0.05553 (3)	−0.08906 (3)	0.15325 (3)	0.02152 (10)	
Cu3	−0.21068 (3)	−0.20781 (3)	−0.02174 (3)	0.02028 (10)	
Cu4	0.12605 (3)	0.09692 (3)	0.22732 (3)	0.02211 (10)	
Cu5	0.31920 (3)	0.29053 (3)	0.24280 (3)	0.02350 (10)	
Cl1	−0.03648 (6)	−0.25637 (6)	0.02371 (6)	0.02707 (17)	
Cl1S	0.58106 (12)	0.06358 (12)	0.39647 (13)	0.0750 (4)	
Cl2	−0.05437 (6)	0.13306 (6)	0.19807 (6)	0.03094 (18)	
Cl2S	0.5393 (2)	0.2027 (2)	0.56923 (14)	0.1182 (8)	
Cl3	0.48127 (6)	0.26149 (7)	0.27012 (7)	0.0354 (2)	
Cl3S	0.69969 (18)	0.26565 (18)	0.46028 (19)	0.1261 (10)	
O1	0.12609 (16)	0.06840 (17)	−0.03527 (15)	0.0207 (4)	
O2	0.07482 (16)	−0.01795 (17)	0.10925 (15)	0.0204 (4)	
O3	0.18815 (17)	0.20480 (17)	0.15822 (15)	0.0224 (4)	
N1	0.2395 (2)	−0.2690 (2)	−0.1084 (2)	0.0333 (7)	
N2	0.1213 (3)	−0.3829 (2)	−0.2101 (3)	0.0382 (7)	
N3	−0.1984 (2)	−0.1346 (2)	0.18140 (19)	0.0233 (5)	
N4	−0.2699 (2)	−0.1792 (2)	0.10182 (19)	0.0230 (5)	
N5	−0.2919 (2)	−0.3463 (2)	−0.07161 (19)	0.0242 (5)	
N6	−0.3505 (2)	−0.3729 (2)	−0.1587 (2)	0.0275 (6)	
N7	0.0389 (2)	−0.0921 (2)	0.25950 (18)	0.0245 (5)	
N8	0.1245 (2)	−0.0138 (2)	0.28730 (19)	0.0261 (6)	
N9	0.1711 (2)	0.2034 (2)	0.34688 (19)	0.0276 (6)	
N10	0.2632 (2)	0.2713 (2)	0.35634 (19)	0.0286 (6)	
C1	0.1374 (3)	−0.3016 (3)	−0.1335 (3)	0.0325 (7)	
H1	0.0839	−0.2717	−0.1019	0.039*	
C1S	0.5763 (4)	0.1875 (4)	0.4541 (4)	0.0577 (12)	
H1S	0.5235	0.2099	0.4166	0.069*	
C2	0.2919 (3)	−0.3328 (3)	−0.1725 (4)	0.0479 (11)	
H2	0.3658	−0.3274	−0.1719	0.058*	
C3	0.2187 (4)	−0.4027 (3)	−0.2345 (4)	0.0482 (11)	
H3	0.2310	−0.4564	−0.2862	0.058*	
C4	0.2842 (3)	−0.1802 (3)	−0.0298 (3)	0.0331 (8)	
C5	0.2759 (3)	−0.1873 (3)	0.0623 (3)	0.0346 (8)	
C6	0.3152 (3)	−0.0996 (3)	0.1372 (3)	0.0419 (9)	
H6	0.3110	−0.1014	0.2010	0.050*	
C7	0.3607 (3)	−0.0095 (3)	0.1198 (4)	0.0453 (10)	
H7	0.3868	0.0498	0.1716	0.054*	
C8	0.3681 (3)	−0.0060 (3)	0.0277 (4)	0.0431 (10)	
H8	0.3999	0.0560	0.0171	0.052*	
C9	0.3301 (3)	−0.0911 (3)	−0.0504 (3)	0.0379 (9)	
C10	0.2289 (3)	−0.2845 (3)	0.0825 (3)	0.0404 (9)	
H10A	0.2328	−0.2733	0.1515	0.061*	
H10B	0.2681	−0.3376	0.0543	0.061*	
H10C	0.1553	−0.3061	0.0549	0.061*	
C11	0.3389 (3)	−0.0871 (3)	−0.1505 (3)	0.0422 (10)	
H11A	0.3619	−0.0162	−0.1502	0.063*	
H11B	0.2703	−0.1150	−0.1883	0.063*	
H11C	0.3902	−0.1274	−0.1785	0.063*	
C12	0.0168 (3)	−0.4342 (3)	−0.2573 (3)	0.0431 (10)	
C13	−0.0492 (3)	−0.4885 (3)	−0.2096 (4)	0.0451 (10)	
C14	−0.1525 (4)	−0.5298 (4)	−0.2519 (4)	0.0609 (14)	
H14	−0.1999	−0.5663	−0.2209	0.073*	
C15	−0.1856 (5)	−0.5181 (5)	−0.3371 (5)	0.0722 (17)	
H15	−0.2563	−0.5458	−0.3640	0.087*	
C16	−0.1196 (5)	−0.4675 (4)	−0.3842 (4)	0.0621 (14)	
H16	−0.1442	−0.4626	−0.4446	0.075*	
C17	−0.0149 (5)	−0.4219 (4)	−0.3449 (3)	0.0561 (12)	
C18	−0.0148 (3)	−0.5039 (3)	−0.1173 (3)	0.0458 (10)	
H18A	0.0605	−0.5051	−0.1136	0.069*	
H18B	−0.0541	−0.5687	−0.1129	0.069*	
H18C	−0.0281	−0.4480	−0.0648	0.069*	
C19	0.0585 (6)	−0.3621 (4)	−0.3931 (4)	0.0699 (16)	
H19A	0.0828	−0.2926	−0.3508	0.105*	
H19B	0.0218	−0.3595	−0.4522	0.105*	
H19C	0.1190	−0.3948	−0.4078	0.105*	
C20	−0.1967 (3)	−0.0718 (3)	0.3572 (3)	0.0380 (8)	
H20A	−0.1207	−0.0679	0.3562	0.057*	
H20B	−0.2223	−0.1112	0.3999	0.057*	
H20C	−0.2112	−0.0029	0.3800	0.057*	
C21	−0.2511 (3)	−0.1228 (3)	0.2588 (2)	0.0277 (7)	
C22	−0.3577 (3)	−0.1608 (3)	0.2292 (3)	0.0315 (7)	
H22	−0.4131	−0.1630	0.2685	0.038*	
C23	−0.3662 (3)	−0.1947 (3)	0.1306 (3)	0.0275 (7)	
C24	−0.4627 (3)	−0.2392 (3)	0.0604 (3)	0.0351 (8)	
H24A	−0.4782	−0.1884	0.0298	0.053*	
H24B	−0.5223	−0.2589	0.0937	0.053*	
H24C	−0.4506	−0.2993	0.0120	0.053*	
C25	−0.2711 (3)	−0.4173 (3)	0.0651 (3)	0.0337 (8)	
H25A	−0.1993	−0.3775	0.0773	0.051*	
H25B	−0.2696	−0.4868	0.0671	0.051*	
H25C	−0.3132	−0.3856	0.1138	0.051*	
C26	−0.3189 (3)	−0.4206 (3)	−0.0316 (2)	0.0288 (7)	
C27	−0.3961 (3)	−0.4963 (3)	−0.0922 (3)	0.0344 (8)	
H27	−0.4296	−0.5579	−0.0818	0.041*	
C28	−0.4141 (3)	−0.4634 (3)	−0.1711 (3)	0.0337 (8)	
C29	−0.4879 (3)	−0.5158 (3)	−0.2602 (3)	0.0438 (10)	
H29A	−0.5283	−0.4676	−0.2748	0.066*	
H29B	−0.5360	−0.5736	−0.2507	0.066*	
H29C	−0.4475	−0.5403	−0.3132	0.066*	
C30	−0.0271 (3)	−0.2591 (3)	0.2858 (3)	0.0340 (8)	
H30A	−0.0883	−0.2443	0.3193	0.051*	
H30B	0.0039	−0.3083	0.3092	0.051*	
H30C	−0.0491	−0.2877	0.2174	0.051*	
C31	0.0523 (3)	−0.1631 (3)	0.3035 (2)	0.0289 (7)	
C32	0.1479 (3)	−0.1288 (3)	0.3615 (2)	0.0337 (8)	
H32	0.1778	−0.1626	0.4012	0.040*	
C33	0.1901 (3)	−0.0362 (3)	0.3493 (2)	0.0313 (7)	
C34	0.2932 (3)	0.0341 (4)	0.3925 (3)	0.0416 (9)	
H34A	0.3275	0.0582	0.3431	0.062*	
H34B	0.3386	−0.0027	0.4196	0.062*	
H34C	0.2806	0.0924	0.4427	0.062*	
C35	0.0410 (3)	0.1482 (3)	0.4489 (3)	0.0382 (9)	
H35A	−0.0100	0.1295	0.3922	0.057*	
H35B	0.0109	0.1862	0.5041	0.057*	
H35C	0.0579	0.0863	0.4604	0.057*	
C36	0.1390 (3)	0.2132 (3)	0.4339 (2)	0.0343 (8)	
C37	0.2120 (3)	0.2885 (3)	0.5009 (3)	0.0398 (9)	
H37	0.2099	0.3111	0.5678	0.048*	
C38	0.2883 (3)	0.3232 (3)	0.4489 (3)	0.0363 (8)	
C39	0.3843 (3)	0.4062 (3)	0.4831 (3)	0.0440 (10)	
H39A	0.4390	0.3913	0.4410	0.066*	
H39B	0.4102	0.4099	0.5481	0.066*	
H39C	0.3663	0.4716	0.4825	0.066*	
H1H	0.164 (3)	0.022 (3)	−0.056 (3)	0.046 (13)*	
H2H	0.116 (3)	−0.062 (3)	0.093 (3)	0.048 (13)*	
H3H	0.138 (2)	0.240 (3)	0.167 (3)	0.037 (11)*	

### Atomic displacement parameters (Å^2^)

**Table:**

	*U*^11^	*U*^22^	*U*^33^	*U*^12^	*U*^13^	*U*^23^
Cu1	0.0149 (2)	0.0265 (3)	0.0172 (2)	0.00159 (19)	0.00120 (18)	0.0100 (2)
Cu2	0.01761 (18)	0.0302 (2)	0.01721 (18)	0.00066 (14)	0.00122 (13)	0.01019 (15)
Cu3	0.01672 (18)	0.0249 (2)	0.01895 (18)	0.00097 (14)	0.00124 (14)	0.00801 (14)
Cu4	0.01850 (19)	0.0300 (2)	0.01643 (18)	−0.00035 (15)	−0.00013 (14)	0.00803 (15)
Cu5	0.01768 (19)	0.0313 (2)	0.01977 (19)	−0.00124 (15)	0.00073 (14)	0.00850 (15)
Cl1	0.0212 (4)	0.0322 (4)	0.0274 (4)	0.0086 (3)	0.0025 (3)	0.0061 (3)
Cl1S	0.0628 (8)	0.0700 (9)	0.0905 (11)	0.0234 (7)	0.0047 (7)	0.0150 (8)
Cl2	0.0218 (4)	0.0320 (4)	0.0335 (4)	0.0060 (3)	−0.0016 (3)	0.0012 (3)
Cl2S	0.173 (2)	0.1406 (19)	0.0623 (10)	0.0689 (18)	0.0352 (12)	0.0370 (11)
Cl3	0.0200 (4)	0.0500 (5)	0.0394 (5)	0.0045 (3)	0.0017 (3)	0.0201 (4)
Cl3S	0.1041 (15)	0.1175 (16)	0.144 (2)	−0.0473 (13)	−0.0505 (14)	0.0720 (15)
O1	0.0169 (10)	0.0260 (11)	0.0190 (10)	0.0028 (8)	0.0025 (8)	0.0071 (8)
O2	0.0169 (10)	0.0276 (11)	0.0180 (10)	0.0051 (8)	0.0012 (8)	0.0085 (8)
O3	0.0174 (10)	0.0290 (11)	0.0197 (10)	0.0022 (8)	0.0016 (8)	0.0066 (9)
N1	0.0265 (15)	0.0283 (15)	0.0508 (19)	0.0098 (12)	0.0169 (13)	0.0154 (13)
N2	0.0369 (17)	0.0313 (16)	0.0483 (19)	0.0121 (13)	0.0169 (15)	0.0089 (14)
N3	0.0204 (13)	0.0275 (13)	0.0226 (13)	0.0037 (10)	0.0039 (10)	0.0084 (10)
N4	0.0189 (12)	0.0273 (13)	0.0237 (13)	0.0027 (10)	0.0029 (10)	0.0096 (10)
N5	0.0215 (13)	0.0264 (13)	0.0243 (13)	0.0020 (10)	0.0012 (10)	0.0087 (11)
N6	0.0257 (14)	0.0289 (14)	0.0243 (13)	−0.0015 (11)	−0.0022 (11)	0.0068 (11)
N7	0.0237 (13)	0.0311 (14)	0.0194 (12)	0.0045 (11)	0.0018 (10)	0.0087 (11)
N8	0.0204 (13)	0.0392 (16)	0.0198 (12)	0.0020 (11)	−0.0005 (10)	0.0132 (11)
N9	0.0208 (13)	0.0370 (15)	0.0211 (13)	−0.0035 (11)	0.0026 (10)	0.0077 (11)
N10	0.0207 (13)	0.0416 (16)	0.0188 (13)	−0.0040 (11)	−0.0002 (10)	0.0076 (12)
C1	0.0289 (17)	0.0285 (17)	0.042 (2)	0.0083 (14)	0.0122 (15)	0.0099 (15)
C1S	0.060 (3)	0.069 (3)	0.045 (3)	0.007 (2)	−0.002 (2)	0.022 (2)
C2	0.034 (2)	0.039 (2)	0.080 (3)	0.0155 (17)	0.032 (2)	0.022 (2)
C3	0.046 (2)	0.037 (2)	0.065 (3)	0.0177 (18)	0.031 (2)	0.009 (2)
C4	0.0171 (15)	0.0279 (17)	0.058 (2)	0.0074 (12)	0.0085 (15)	0.0162 (16)
C5	0.0192 (16)	0.0327 (18)	0.056 (2)	0.0090 (13)	0.0086 (15)	0.0170 (17)
C6	0.0264 (18)	0.041 (2)	0.060 (3)	0.0095 (15)	0.0075 (17)	0.0162 (19)
C7	0.0251 (18)	0.036 (2)	0.071 (3)	0.0043 (15)	0.0041 (18)	0.010 (2)
C8	0.0215 (17)	0.0289 (18)	0.081 (3)	0.0027 (14)	0.0108 (18)	0.0199 (19)
C9	0.0167 (15)	0.0355 (19)	0.067 (3)	0.0070 (13)	0.0086 (16)	0.0221 (18)
C10	0.0251 (18)	0.040 (2)	0.063 (3)	0.0069 (15)	0.0097 (17)	0.0256 (19)
C11	0.0270 (18)	0.039 (2)	0.070 (3)	0.0070 (15)	0.0157 (18)	0.029 (2)
C12	0.042 (2)	0.035 (2)	0.045 (2)	0.0147 (17)	0.0047 (18)	−0.0048 (17)
C13	0.037 (2)	0.0289 (19)	0.061 (3)	0.0051 (16)	0.0069 (19)	−0.0008 (18)
C14	0.042 (3)	0.047 (3)	0.081 (4)	0.005 (2)	0.000 (2)	0.000 (2)
C15	0.060 (3)	0.064 (3)	0.074 (4)	0.012 (3)	−0.011 (3)	−0.007 (3)
C16	0.073 (4)	0.056 (3)	0.045 (3)	0.024 (3)	−0.009 (2)	−0.008 (2)
C17	0.079 (4)	0.046 (3)	0.040 (2)	0.026 (2)	0.012 (2)	−0.0018 (19)
C18	0.038 (2)	0.033 (2)	0.061 (3)	0.0022 (16)	0.0105 (19)	0.0081 (19)
C19	0.110 (5)	0.059 (3)	0.043 (3)	0.034 (3)	0.026 (3)	0.005 (2)
C20	0.040 (2)	0.050 (2)	0.0229 (17)	0.0071 (17)	0.0109 (15)	0.0089 (15)
C21	0.0279 (16)	0.0328 (17)	0.0256 (16)	0.0053 (13)	0.0100 (13)	0.0126 (13)
C22	0.0265 (17)	0.0393 (19)	0.0333 (18)	0.0085 (14)	0.0146 (14)	0.0147 (15)
C23	0.0212 (15)	0.0284 (16)	0.0372 (18)	0.0063 (12)	0.0072 (13)	0.0151 (14)
C24	0.0214 (16)	0.039 (2)	0.046 (2)	0.0012 (14)	0.0024 (15)	0.0183 (17)
C25	0.0359 (19)	0.0342 (18)	0.0343 (18)	0.0041 (15)	0.0006 (15)	0.0179 (15)
C26	0.0277 (16)	0.0294 (17)	0.0310 (17)	0.0034 (13)	0.0041 (13)	0.0125 (14)
C27	0.0365 (19)	0.0267 (17)	0.0384 (19)	−0.0031 (14)	0.0000 (15)	0.0132 (15)
C28	0.0325 (18)	0.0328 (18)	0.0308 (18)	−0.0035 (14)	−0.0009 (14)	0.0078 (14)
C29	0.045 (2)	0.038 (2)	0.037 (2)	−0.0138 (17)	−0.0060 (17)	0.0070 (16)
C30	0.048 (2)	0.0302 (18)	0.0281 (17)	0.0093 (15)	0.0075 (15)	0.0132 (14)
C31	0.0322 (18)	0.0386 (19)	0.0224 (15)	0.0128 (14)	0.0096 (13)	0.0148 (14)
C32	0.0306 (18)	0.053 (2)	0.0265 (17)	0.0141 (16)	0.0064 (14)	0.0209 (16)
C33	0.0230 (16)	0.052 (2)	0.0234 (16)	0.0088 (15)	0.0029 (12)	0.0181 (15)
C34	0.0228 (17)	0.073 (3)	0.0309 (19)	0.0028 (17)	−0.0053 (14)	0.0239 (19)
C35	0.0293 (18)	0.054 (2)	0.0231 (16)	−0.0087 (16)	0.0082 (14)	0.0076 (16)
C36	0.0277 (17)	0.046 (2)	0.0236 (16)	−0.0059 (15)	0.0033 (13)	0.0084 (15)
C37	0.037 (2)	0.051 (2)	0.0203 (16)	−0.0094 (17)	0.0042 (14)	0.0044 (15)
C38	0.0305 (18)	0.045 (2)	0.0236 (16)	−0.0061 (15)	−0.0002 (14)	0.0036 (15)
C39	0.038 (2)	0.053 (2)	0.0255 (18)	−0.0155 (18)	−0.0030 (15)	0.0027 (17)

### Geometric parameters (Å, º)

**Table:**

Cu1—O1^i^	1.924 (2)	C9—C11	1.500 (6)
Cu1—O1	1.924 (2)	C10—H10A	0.9800
Cu1—O2	1.929 (2)	C10—H10B	0.9800
Cu1—O2^i^	1.929 (2)	C10—H10C	0.9800
Cu1—Cu2^i^	2.9293 (4)	C11—H11A	0.9800
Cu1—Cu2	2.9293 (4)	C11—H11B	0.9800
Cu2—N7	1.947 (3)	C11—H11C	0.9800
Cu2—N3	1.964 (3)	C12—C13	1.393 (7)
Cu2—O1^i^	2.031 (2)	C12—C17	1.397 (7)
Cu2—O2	2.044 (2)	C13—C14	1.401 (6)
Cu2—Cl1	2.6282 (9)	C13—C18	1.489 (7)
Cu2—Cu3	3.0558 (5)	C14—C15	1.364 (9)
Cu3—N5	1.950 (3)	C14—H14	0.9500
Cu3—N4	1.979 (3)	C15—C16	1.360 (9)
Cu3—O1^i^	1.986 (2)	C15—H15	0.9500
Cu3—O3^i^	2.057 (2)	C16—C17	1.414 (8)
Cu3—Cl1	2.5755 (8)	C16—H16	0.9500
Cu4—N9	1.943 (3)	C17—C19	1.497 (8)
Cu4—N8	1.977 (3)	C18—H18A	0.9800
Cu4—O2	1.996 (2)	C18—H18B	0.9800
Cu4—O3	2.115 (2)	C18—H18C	0.9800
Cu4—Cl2	2.5191 (9)	C19—H19A	0.9800
Cu5—N6^i^	1.924 (3)	C19—H19B	0.9800
Cu5—N10	1.927 (3)	C19—H19C	0.9800
Cu5—O3	2.029 (2)	C20—C21	1.498 (5)
Cu5—Cl3	2.2466 (9)	C20—H20A	0.9800
Cl1S—C1S	1.712 (6)	C20—H20B	0.9800
Cl2S—C1S	1.753 (5)	C20—H20C	0.9800
Cl3S—C1S	1.760 (6)	C21—C22	1.394 (5)
O1—Cu3^i^	1.986 (2)	C22—C23	1.388 (5)
O1—Cu2^i^	2.031 (2)	C22—H22	0.9500
O1—H1H	0.874 (10)	C23—C24	1.501 (5)
O2—H2H	0.877 (10)	C24—H24A	0.9800
O3—Cu3^i^	2.057 (2)	C24—H24B	0.9800
O3—H3H	0.876 (10)	C24—H24C	0.9800
N1—C1	1.322 (5)	C25—C26	1.505 (5)
N1—C2	1.399 (5)	C25—H25A	0.9800
N1—C4	1.446 (5)	C25—H25B	0.9800
N2—C1	1.334 (5)	C25—H25C	0.9800
N2—C3	1.386 (5)	C26—C27	1.389 (5)
N2—C12	1.457 (5)	C27—C28	1.384 (5)
N3—C21	1.347 (4)	C27—H27	0.9500
N3—N4	1.377 (4)	C28—C29	1.503 (5)
N4—C23	1.346 (4)	C29—H29A	0.9800
N5—C26	1.335 (4)	C29—H29B	0.9800
N5—N6	1.375 (4)	C29—H29C	0.9800
N6—C28	1.344 (4)	C30—C31	1.493 (5)
N6—Cu5^i^	1.924 (3)	C30—H30A	0.9800
N7—C31	1.352 (4)	C30—H30B	0.9800
N7—N8	1.371 (4)	C30—H30C	0.9800
N8—C33	1.349 (4)	C31—C32	1.397 (5)
N9—C36	1.347 (4)	C32—C33	1.376 (5)
N9—N10	1.368 (4)	C32—H32	0.9500
N10—C38	1.343 (4)	C33—C34	1.506 (5)
C1—H1	0.9500	C34—H34A	0.9800
C1S—H1S	1.0000	C34—H34B	0.9800
C2—C3	1.334 (7)	C34—H34C	0.9800
C2—H2	0.9500	C35—C36	1.498 (5)
C3—H3	0.9500	C35—H35A	0.9800
C4—C5	1.394 (6)	C35—H35B	0.9800
C4—C9	1.402 (5)	C35—H35C	0.9800
C5—C6	1.394 (6)	C36—C37	1.397 (5)
C5—C10	1.503 (5)	C37—C38	1.390 (5)
C6—C7	1.390 (6)	C37—H37	0.9500
C6—H6	0.9500	C38—C39	1.504 (5)
C7—C8	1.378 (7)	C39—H39A	0.9800
C7—H7	0.9500	C39—H39B	0.9800
C8—C9	1.396 (6)	C39—H39C	0.9800
C8—H8	0.9500		
			
O1^i^—Cu1—O1	180.0	C7—C6—H6	119.6
O1^i^—Cu1—O2	87.68 (9)	C5—C6—H6	119.6
O1—Cu1—O2	92.32 (9)	C8—C7—C6	120.2 (4)
O1^i^—Cu1—O2^i^	92.32 (9)	C8—C7—H7	119.9
O1—Cu1—O2^i^	87.68 (9)	C6—C7—H7	119.9
O2—Cu1—O2^i^	180.0	C7—C8—C9	121.8 (4)
O1^i^—Cu1—Cu2^i^	136.36 (6)	C7—C8—H8	119.1
O1—Cu1—Cu2^i^	43.64 (6)	C9—C8—H8	119.1
O2—Cu1—Cu2^i^	135.95 (6)	C8—C9—C4	116.2 (4)
O2^i^—Cu1—Cu2^i^	44.05 (6)	C8—C9—C11	121.6 (4)
O1^i^—Cu1—Cu2	43.64 (6)	C4—C9—C11	122.2 (4)
O1—Cu1—Cu2	136.36 (6)	C5—C10—H10A	109.5
O2—Cu1—Cu2	44.05 (6)	C5—C10—H10B	109.5
O2^i^—Cu1—Cu2	135.95 (6)	H10A—C10—H10B	109.5
Cu2^i^—Cu1—Cu2	180.0	C5—C10—H10C	109.5
N7—Cu2—N3	106.25 (11)	H10A—C10—H10C	109.5
N7—Cu2—O1^i^	167.65 (10)	H10B—C10—H10C	109.5
N3—Cu2—O1^i^	85.78 (10)	C9—C11—H11A	109.5
N7—Cu2—O2	85.87 (10)	C9—C11—H11B	109.5
N3—Cu2—O2	165.28 (10)	H11A—C11—H11B	109.5
O1^i^—Cu2—O2	81.83 (8)	C9—C11—H11C	109.5
N7—Cu2—Cl1	101.13 (9)	H11A—C11—H11C	109.5
N3—Cu2—Cl1	98.45 (8)	H11B—C11—H11C	109.5
O1^i^—Cu2—Cl1	79.34 (7)	C13—C12—C17	123.0 (4)
O2—Cu2—Cl1	87.07 (7)	C13—C12—N2	118.0 (4)
N7—Cu2—Cu1	126.85 (8)	C17—C12—N2	118.9 (4)
N3—Cu2—Cu1	126.06 (8)	C12—C13—C14	117.4 (5)
O1^i^—Cu2—Cu1	40.83 (6)	C12—C13—C18	123.1 (4)
O2—Cu2—Cu1	41.01 (6)	C14—C13—C18	119.5 (5)
Cl1—Cu2—Cu1	81.59 (2)	C15—C14—C13	120.7 (6)
N7—Cu2—Cu3	148.15 (8)	C15—C14—H14	119.6
N3—Cu2—Cu3	65.40 (8)	C13—C14—H14	119.6
O1^i^—Cu2—Cu3	39.93 (6)	C16—C15—C14	121.4 (5)
O2—Cu2—Cu3	108.52 (6)	C16—C15—H15	119.3
Cl1—Cu2—Cu3	53.243 (19)	C14—C15—H15	119.3
Cu1—Cu2—Cu3	72.926 (12)	C15—C16—C17	121.0 (5)
N5—Cu3—N4	95.03 (11)	C15—C16—H16	119.5
N5—Cu3—O1^i^	177.04 (10)	C17—C16—H16	119.5
N4—Cu3—O1^i^	82.97 (10)	C12—C17—C16	116.5 (5)
N5—Cu3—O3^i^	89.83 (10)	C12—C17—C19	121.1 (5)
N4—Cu3—O3^i^	158.66 (10)	C16—C17—C19	122.4 (5)
O1^i^—Cu3—O3^i^	92.81 (9)	C13—C18—H18A	109.5
N5—Cu3—Cl1	96.83 (8)	C13—C18—H18B	109.5
N4—Cu3—Cl1	101.02 (8)	H18A—C18—H18B	109.5
O1^i^—Cu3—Cl1	81.45 (6)	C13—C18—H18C	109.5
O3^i^—Cu3—Cl1	99.01 (6)	H18A—C18—H18C	109.5
N5—Cu3—Cu2	136.06 (8)	H18B—C18—H18C	109.5
N4—Cu3—Cu2	63.99 (8)	C17—C19—H19A	109.5
O1^i^—Cu3—Cu2	41.03 (6)	C17—C19—H19B	109.5
O3^i^—Cu3—Cu2	124.15 (6)	H19A—C19—H19B	109.5
Cl1—Cu3—Cu2	54.84 (2)	C17—C19—H19C	109.5
N9—Cu4—N8	93.34 (12)	H19A—C19—H19C	109.5
N9—Cu4—O2	176.28 (11)	H19B—C19—H19C	109.5
N8—Cu4—O2	83.51 (10)	C21—C20—H20A	109.5
N9—Cu4—O3	87.28 (10)	C21—C20—H20B	109.5
N8—Cu4—O3	157.45 (10)	H20A—C20—H20B	109.5
O2—Cu4—O3	96.39 (9)	C21—C20—H20C	109.5
N9—Cu4—Cl2	97.75 (9)	H20A—C20—H20C	109.5
N8—Cu4—Cl2	112.62 (8)	H20B—C20—H20C	109.5
O2—Cu4—Cl2	81.71 (6)	N3—C21—C22	108.7 (3)
O3—Cu4—Cl2	89.57 (6)	N3—C21—C20	121.8 (3)
N6^i^—Cu5—N10	150.43 (14)	C22—C21—C20	129.4 (3)
N6^i^—Cu5—O3	93.11 (10)	C23—C22—C21	105.8 (3)
N10—Cu5—O3	92.56 (10)	C23—C22—H22	127.1
N6^i^—Cu5—Cl3	98.22 (9)	C21—C22—H22	127.1
N10—Cu5—Cl3	98.93 (9)	N4—C23—C22	109.1 (3)
O3—Cu5—Cl3	133.07 (7)	N4—C23—C24	121.5 (3)
Cu3—Cl1—Cu2	71.91 (2)	C22—C23—C24	129.4 (3)
Cu1—O1—Cu3^i^	131.01 (12)	C23—C24—H24A	109.5
Cu1—O1—Cu2^i^	95.53 (9)	C23—C24—H24B	109.5
Cu3^i^—O1—Cu2^i^	99.04 (9)	H24A—C24—H24B	109.5
Cu1—O1—H1H	107 (3)	C23—C24—H24C	109.5
Cu3^i^—O1—H1H	114 (3)	H24A—C24—H24C	109.5
Cu2^i^—O1—H1H	106 (3)	H24B—C24—H24C	109.5
Cu1—O2—Cu4	122.22 (11)	C26—C25—H25A	109.5
Cu1—O2—Cu2	94.94 (9)	C26—C25—H25B	109.5
Cu4—O2—Cu2	99.60 (9)	H25A—C25—H25B	109.5
Cu1—O2—H2H	112 (3)	C26—C25—H25C	109.5
Cu4—O2—H2H	116 (3)	H25A—C25—H25C	109.5
Cu2—O2—H2H	107 (3)	H25B—C25—H25C	109.5
Cu5—O3—Cu3^i^	106.67 (10)	N5—C26—C27	109.2 (3)
Cu5—O3—Cu4	105.71 (9)	N5—C26—C25	123.0 (3)
Cu3^i^—O3—Cu4	137.76 (11)	C27—C26—C25	127.8 (3)
Cu5—O3—H3H	108 (3)	C28—C27—C26	105.7 (3)
Cu3^i^—O3—H3H	98 (3)	C28—C27—H27	127.2
Cu4—O3—H3H	97 (3)	C26—C27—H27	127.2
C1—N1—C2	107.9 (3)	N6—C28—C27	108.8 (3)
C1—N1—C4	123.7 (3)	N6—C28—C29	122.2 (3)
C2—N1—C4	128.3 (3)	C27—C28—C29	129.0 (3)
C1—N2—C3	107.8 (3)	C28—C29—H29A	109.5
C1—N2—C12	122.7 (3)	C28—C29—H29B	109.5
C3—N2—C12	129.4 (4)	H29A—C29—H29B	109.5
C21—N3—N4	108.3 (3)	C28—C29—H29C	109.5
C21—N3—Cu2	137.0 (2)	H29A—C29—H29C	109.5
N4—N3—Cu2	114.0 (2)	H29B—C29—H29C	109.5
C23—N4—N3	108.1 (3)	C31—C30—H30A	109.5
C23—N4—Cu3	135.9 (2)	C31—C30—H30B	109.5
N3—N4—Cu3	115.96 (19)	H30A—C30—H30B	109.5
C26—N5—N6	108.1 (3)	C31—C30—H30C	109.5
C26—N5—Cu3	132.5 (2)	H30A—C30—H30C	109.5
N6—N5—Cu3	118.3 (2)	H30B—C30—H30C	109.5
C28—N6—N5	108.3 (3)	N7—C31—C32	108.1 (3)
C28—N6—Cu5^i^	132.2 (2)	N7—C31—C30	121.4 (3)
N5—N6—Cu5^i^	119.0 (2)	C32—C31—C30	130.5 (3)
C31—N7—N8	108.7 (3)	C33—C32—C31	106.0 (3)
C31—N7—Cu2	134.7 (2)	C33—C32—H32	127.0
N8—N7—Cu2	115.3 (2)	C31—C32—H32	127.0
C33—N8—N7	107.7 (3)	N8—C33—C32	109.5 (3)
C33—N8—Cu4	136.1 (2)	N8—C33—C34	121.2 (3)
N7—N8—Cu4	116.14 (19)	C32—C33—C34	129.3 (3)
C36—N9—N10	108.3 (3)	C33—C34—H34A	109.5
C36—N9—Cu4	130.7 (2)	C33—C34—H34B	109.5
N10—N9—Cu4	119.5 (2)	H34A—C34—H34B	109.5
C38—N10—N9	108.5 (3)	C33—C34—H34C	109.5
C38—N10—Cu5	132.3 (2)	H34A—C34—H34C	109.5
N9—N10—Cu5	118.5 (2)	H34B—C34—H34C	109.5
N1—C1—N2	109.5 (3)	C36—C35—H35A	109.5
N1—C1—H1	125.3	C36—C35—H35B	109.5
N2—C1—H1	125.3	H35A—C35—H35B	109.5
Cl1S—C1S—Cl2S	112.4 (3)	C36—C35—H35C	109.5
Cl1S—C1S—Cl3S	109.8 (3)	H35A—C35—H35C	109.5
Cl2S—C1S—Cl3S	109.4 (3)	H35B—C35—H35C	109.5
Cl1S—C1S—H1S	108.4	N9—C36—C37	108.8 (3)
Cl2S—C1S—H1S	108.4	N9—C36—C35	122.0 (3)
Cl3S—C1S—H1S	108.4	C37—C36—C35	129.2 (3)
C3—C2—N1	107.1 (4)	C38—C37—C36	105.3 (3)
C3—C2—H2	126.4	C38—C37—H37	127.3
N1—C2—H2	126.4	C36—C37—H37	127.3
C2—C3—N2	107.8 (4)	N10—C38—C37	109.1 (3)
C2—C3—H3	126.1	N10—C38—C39	121.8 (3)
N2—C3—H3	126.1	C37—C38—C39	129.1 (3)
C5—C4—C9	123.8 (4)	C38—C39—H39A	109.5
C5—C4—N1	117.9 (3)	C38—C39—H39B	109.5
C9—C4—N1	118.2 (4)	H39A—C39—H39B	109.5
C4—C5—C6	117.1 (4)	C38—C39—H39C	109.5
C4—C5—C10	122.8 (4)	H39A—C39—H39C	109.5
C6—C5—C10	120.0 (4)	H39B—C39—H39C	109.5
C7—C6—C5	120.8 (4)		
			
C21—N3—N4—C23	0.1 (4)	C13—C12—C17—C16	0.2 (6)
Cu2—N3—N4—C23	−172.1 (2)	N2—C12—C17—C16	−175.7 (4)
C21—N3—N4—Cu3	−178.5 (2)	C13—C12—C17—C19	179.4 (4)
Cu2—N3—N4—Cu3	9.3 (3)	N2—C12—C17—C19	3.5 (6)
C26—N5—N6—C28	0.6 (4)	C15—C16—C17—C12	1.7 (7)
Cu3—N5—N6—C28	−168.8 (2)	C15—C16—C17—C19	−177.5 (5)
C26—N5—N6—Cu5^i^	−172.4 (2)	N4—N3—C21—C22	0.4 (4)
Cu3—N5—N6—Cu5^i^	18.2 (3)	Cu2—N3—C21—C22	169.8 (3)
C31—N7—N8—C33	−0.7 (4)	N4—N3—C21—C20	−177.2 (3)
Cu2—N7—N8—C33	−169.7 (2)	Cu2—N3—C21—C20	−7.7 (5)
C31—N7—N8—Cu4	177.2 (2)	N3—C21—C22—C23	−0.6 (4)
Cu2—N7—N8—Cu4	8.2 (3)	C20—C21—C22—C23	176.7 (4)
C36—N9—N10—C38	0.0 (4)	N3—N4—C23—C22	−0.5 (4)
Cu4—N9—N10—C38	−167.5 (3)	Cu3—N4—C23—C22	177.7 (3)
C36—N9—N10—Cu5	−171.0 (3)	N3—N4—C23—C24	177.8 (3)
Cu4—N9—N10—Cu5	21.4 (4)	Cu3—N4—C23—C24	−4.0 (5)
C2—N1—C1—N2	0.1 (4)	C21—C22—C23—N4	0.7 (4)
C4—N1—C1—N2	178.1 (3)	C21—C22—C23—C24	−177.4 (3)
C3—N2—C1—N1	0.0 (5)	N6—N5—C26—C27	−0.4 (4)
C12—N2—C1—N1	−177.6 (4)	Cu3—N5—C26—C27	167.0 (3)
C1—N1—C2—C3	−0.2 (5)	N6—N5—C26—C25	−179.5 (3)
C4—N1—C2—C3	−178.1 (4)	Cu3—N5—C26—C25	−12.2 (5)
N1—C2—C3—N2	0.2 (5)	N5—C26—C27—C28	0.0 (4)
C1—N2—C3—C2	−0.2 (5)	C25—C26—C27—C28	179.1 (4)
C12—N2—C3—C2	177.2 (4)	N5—N6—C28—C27	−0.6 (4)
C1—N1—C4—C5	73.0 (5)	Cu5^i^—N6—C28—C27	171.1 (3)
C2—N1—C4—C5	−109.5 (4)	N5—N6—C28—C29	−179.0 (4)
C1—N1—C4—C9	−105.0 (4)	Cu5^i^—N6—C28—C29	−7.3 (6)
C2—N1—C4—C9	72.6 (5)	C26—C27—C28—N6	0.3 (5)
C9—C4—C5—C6	0.5 (5)	C26—C27—C28—C29	178.6 (4)
N1—C4—C5—C6	−177.3 (3)	N8—N7—C31—C32	0.7 (4)
C9—C4—C5—C10	−178.2 (3)	Cu2—N7—C31—C32	166.7 (3)
N1—C4—C5—C10	4.0 (5)	N8—N7—C31—C30	−178.6 (3)
C4—C5—C6—C7	0.0 (5)	Cu2—N7—C31—C30	−12.7 (5)
C10—C5—C6—C7	178.8 (3)	N7—C31—C32—C33	−0.5 (4)
C5—C6—C7—C8	−0.5 (6)	C30—C31—C32—C33	178.8 (4)
C6—C7—C8—C9	0.5 (6)	N7—N8—C33—C32	0.4 (4)
C7—C8—C9—C4	0.0 (5)	Cu4—N8—C33—C32	−176.9 (3)
C7—C8—C9—C11	−179.7 (4)	N7—N8—C33—C34	179.0 (3)
C5—C4—C9—C8	−0.5 (5)	Cu4—N8—C33—C34	1.7 (6)
N1—C4—C9—C8	177.3 (3)	C31—C32—C33—N8	0.0 (4)
C5—C4—C9—C11	179.2 (3)	C31—C32—C33—C34	−178.4 (4)
N1—C4—C9—C11	−3.1 (5)	N10—N9—C36—C37	−0.3 (5)
C1—N2—C12—C13	−69.6 (5)	Cu4—N9—C36—C37	165.3 (3)
C3—N2—C12—C13	113.3 (5)	N10—N9—C36—C35	−178.9 (4)
C1—N2—C12—C17	106.5 (5)	Cu4—N9—C36—C35	−13.3 (6)
C3—N2—C12—C17	−70.6 (6)	N9—C36—C37—C38	0.5 (5)
C17—C12—C13—C14	−1.5 (6)	C35—C36—C37—C38	179.0 (4)
N2—C12—C13—C14	174.4 (4)	N9—N10—C38—C37	0.3 (5)
C17—C12—C13—C18	178.5 (4)	Cu5—N10—C38—C37	169.7 (3)
N2—C12—C13—C18	−5.6 (6)	N9—N10—C38—C39	−178.3 (4)
C12—C13—C14—C15	0.9 (7)	Cu5—N10—C38—C39	−9.0 (6)
C18—C13—C14—C15	−179.0 (5)	C36—C37—C38—N10	−0.5 (5)
C13—C14—C15—C16	0.9 (8)	C36—C37—C38—C39	178.0 (4)
C14—C15—C16—C17	−2.3 (8)		

Symmetry code: (i) −*x*, −*y*, −*z*.

### Hydrogen-bond geometry (Å, º)

**Table:**

*D*—H···*A*	*D*—H	H···*A*	*D*···*A*	*D*—H···*A*
O1—H1*H*···Cl2^i^	0.87 (1)	2.68 (4)	3.098 (2)	111 (3)
O2—H2*H*···Cl1	0.88 (1)	2.95 (5)	3.246 (2)	102 (3)
O3—H3*H*···Cl2	0.88 (1)	2.81 (4)	3.277 (2)	115 (3)
C1—H1···Cl1	0.95	2.48	3.336 (4)	151
C2—H2···Cl3^ii^	0.95	2.71	3.467 (4)	138
C1*S*—H1*S*···Cl3	1.00	2.51	3.395 (5)	147
C20—H20*A*···N7	0.98	2.60	3.477 (5)	149
C24—H24*C*···N5	0.98	2.55	3.307 (5)	134
C25—H25*A*···Cl1	0.98	2.77	3.652 (4)	150
C29—H29*A*···Cl3^i^	0.98	2.81	3.572 (5)	135
C35—H35*A*···Cl2	0.98	2.90	3.764 (4)	148
C39—H39*A*···Cl3	0.98	2.80	3.644 (4)	144

Symmetry codes: (i) −*x*, −*y*, −*z*; (ii) −*x*+1, −*y*, −*z*.
